# Analyzing the Prevalence of Smartphone Addiction (SA) Among Preschoolers (3–6 Years) and Its Demographic and Familial Risk Factors: A Cross‐Sectional Study in Noakhali District, Bangladesh

**DOI:** 10.1155/tswj/6662898

**Published:** 2026-09-17

**Authors:** Kabir Hossain, Md. Mamun Miah, Najma Begum

**Affiliations:** ^1^ Department of Statistics, Noakhali Science and Technology University, Noakhali, Bangladesh, nstu.edu.bd; ^2^ Bangladesh Institute for Research and Statistical Training (BIRST), Dhaka, Bangladesh

**Keywords:** Bangladesh, logistic regression, Noakhali, preschoolers, smartphone addiction (SA)

## Abstract

**Background and Aims:**

Smartphone Addiction (SA) is emerging as a growing public health concern, especially affecting the youth on a global scale. The present study was administered to investigate the prevalence of SA among preschoolers and its demographic and familial influential factors.

**Methods:**

Primary data were collected from 441 preschoolers (3–6 years) using an interviewer‐administered, face‐to‐face interview that collected sociodemographic and familial information from May 10, 2023, to June 25, 2023. Initially, the *χ*2 test was employed to find the association between SA and selected demographic and familial variables. Secondly, the multivariable logistic regression model was applied to determine the impact of the associated variables on SA.

**Results:**

Findings revealed that 69% of the preschoolers were SA with 52.5% male and 47.5% female. The results of logistic regression illustrated that SA was influenced by preschoolers′ fathers′ profession (AOR: 0.270; 95% CI: 0.123, 0.592), owning smartphone (AOR: 0.079; 95% CI: 0.027, 0.230), parents′ positive perception and behavior of using smartphone (AOR: 0.220; 95% CI: 0.052, 0.925), and more time spent at smartphone daily (AOR: 5.416; 95% CI: 2.80, 10.476). Preschoolers with fathers in formal employment, such as a service job, have lower SA than those with fathers in business. Furthermore, preschoolers whose parents have negative attitudes of smartphone use, particularly extremely negative perceptions, have lower SA than those whose parents have a more positive outlook.

**Conclusions:**

Based on the findings of the study, the authors recommend that fathers, particularly those in business, take extra precautions to avoid their children from getting SA. Furthermore, parents should be cautioned against giving their children personal smartphones, recognizing the harmful effects on both mental and physical health. Parents should not let their children use their cellphones for an extended period. To mitigate the negative consequences of smartphone use, parents must provide adequate attention and time to their children. Encouraging children to explore different forms of entertainment other than mobile phones is critical in preventing the development of SA.

## 1. Introduction

Smartphones are innovative, flexible devices that combine mobile phone functionality with personal computer capabilities [1], revolutionizing work, play, and communication in modern life. Smartphone addiction (SA) is a growing concern [2] among researchers, educators, and parents, particularly among vulnerable groups like preschoolers aged 1–5 years [3, 4]. The digital revolution has transformed daily life but also raises concerns about potential negative consequences. As of January 26, 2023, there were 6.5 billion global smartphone users [5].

Bangladesh Bureau of Statistics (BBS) survey reported that Bangladesh has 38.9% internet users, 89.9% mobile phone users, and 30.9% using smartphones. The mobile phone usage at the household level is 52.2% [6]. The majority of youngsters about 96.6% of children regularly use mobile devices. 59% and 61% of United States and Japanese parents believe their children have developed technology addictions, respectively, with 47% believing their children are addicted to smartphones [7]. In Dhaka, Bangladesh, the SA rate among young children is 86% among those aged 3–5. Youngsters who use their smartphones for more than 1 h each day have a higher chance of developing PSU (Problematic Smartphone Use). Parental smartphone use, education, occupation, family income, and the mother′s age were additional crucial variables for developing PSU [8]. According to a cross‐sectional study of young adults in Bangladesh, 61.4% of young adults are addicted to their smartphones [9].

Preschoolers addicted to smartphones face various physical and mental health issues, including ADHD, emotional instability, aggression, depression, lack of control, impaired vision, obesity, body imbalance, and brain development issues [8]. A nationwide survey on smartphone overdependence was conducted in 2017 by Korea; the findings from this study indicate that one in five preschool children using smartphones are associated with PSU compared with other age groups [10]. Preschoolers′ addiction to smartphones is significantly and positively influenced by children′s depression and social withdrawal symptoms [11]. Previously conducted research found that the probability of developing PSU significantly increased with smartphone use to watch TV shows or videos for entertainment [10]. Hyperactive‐distractible child behaviors and low parenting efficacy among mothers significantly influenced the SA of preschoolers [12]. According to a study carried out among preschoolers in Turkey, a child′s level of inactivity and attitude are associated with their interaction with television [13]. A cross‐sectional study reveals that SA among kids and teens in mainland China is a significant issue during the epidemic [14].

The addiction to smartphones among parents has significantly impacted children [15]. Bad behavioral characteristics and poor parenting practices increase addiction to television and other digital media [13]. A study published in the journal Developmental Science suggests that mobile phone usage can negatively impact newborn social and psychological development and parent–child relationships [16]. Young individuals are at a higher risk of developing SA [17] and prior research suggests that SA causes physical issues like absence of physical exercise, fatigue, and joint disorders [18–20]. Younger and younger preschoolers are becoming familiar with smartphones [21] and other digital devices. Excessive screen time is linked to delayed sleep and reduced sleep duration in preschoolers [22].

The existing literature focuses on the prevalence, determinants, and consequences of SA among both preschoolers and young adults, with a specific emphasis on urban regions worldwide. In Bangladesh, few authors tried to analyze the prevalence and risk factors of SA but in the city areas of the country like Dhaka where the internet as well as smartphones are highly available. Because of the technological advancement in Bangladesh, smartphones and internet access are now accessible at the district level and in rural households as well. Therefore, it is crucial to know the factors associated with SA among preschoolers in rural areas like Noakhali, Bangladesh. The present study intends to analyze the prevalence of SA among preschoolers and its demographic and familial risk factors.

## 2. Methods

### 2.1. Study Design and Setting

The study was conducted in Noakhali District, Bangladesh, which is predominantly a rural area with emerging semiurban (rurban) communities. The study targeted preschool children aged 3–6 years residing in the selected communities of the district. Since direct assessment of children′s smartphone use was not feasible, information was obtained from their parents or primary caregivers, who served as proxy respondents. Caregivers were included because they are the primary observers of children′s daily behaviors and smartphone use patterns, and thus can provide reliable information regarding the child′s usage.

#### 2.1.1. Inclusion Criteria

Caregivers were included in the study if they were parents or primary caregivers of preschool children aged 3–6 years residing in the selected areas of Noakhali District. Eligible participants were required to have been living with the child for at least the past 6 months and to be directly responsible for the child′s daily care and monitoring of smartphone use. Only caregivers who provided informed consent and were able to communicate in Bangla were included in the study.

#### 2.1.2. Exclusion Criteria

Caregivers were excluded if they were not responsible for children within the specified age range of 3–6 years or were not the primary caregiver of the child. Individuals who declined to participate or did not provide informed consent were also excluded. In addition, questionnaires with incomplete or inconsistent responses that could compromise data quality were removed from the final analysis.

### 2.2. Sampling Strategy

The study employed a multistage sampling design. In the first stage, Upazilas were selected using random sampling. In the final stage, eligible households were identified within selected clusters. However, a complete sampling frame of households with preschool‐aged children was not available, which precluded probability‐based sampling at the household level. An initial list of households was obtained from administrative records, but it did not permit systematic random selection of eligible households. Therefore, households were identified through field screening and recruited using a convenience sampling approach. This approach may have introduced selection bias and limits the representativeness of the study population. Accordingly, findings should be interpreted with caution regarding their generalizability beyond similar rural‐dominant settings.

### 2.3. Sample Size and Data Collection Procedure

The required sample size for the study was determined based on Roscoe (1975) guideline [23], which suggests that a sample size between 30 and 500 is generally appropriate for most behavioral research. Accordingly, a sample within this range was considered adequate for the present study.

Prior to data collection, participants were informed about the purpose of the study and invited to participate voluntarily. Written or verbal informed consent was obtained from all participants, and they were assured of the confidentiality and anonymity of their responses.

Primary data were collected by four trained undergraduate students. The data collectors underwent a structured training session conducted by the authors to ensure standardization of data collection procedures and minimize interviewer bias. Following training, interviewer‐administered face‐to‐face interviews were conducted between May 10, 2023, and June 25, 2023.

A total of 470 households were approached during the field survey. Among them, 5 households were excluded because they did not own a smartphone, and 29 households were excluded because of the absence of preschool children. Additionally, 6 eligible participants declined to participate. Therefore, the final analytical sample consisted of 441 participants, yielding a response rate of over 95%.

Data were collected using a structured, interviewer‐administered questionnaire to ensure clarity of understanding and to minimize missing or incomplete responses. The questionnaire was developed based on previously published literature on SA and PSU. Content validity was ensured through expert review by public health and statistics professionals, who evaluated and refined the instrument based on their feedback.

Before the main survey, a pilot study was conducted among 30 parents from a neighboring community who were not included in the final sample. The pilot test was used to assess clarity, comprehensibility, and feasibility of the questionnaire, and necessary modifications were made accordingly.

### 2.4. Study Variables and Measurements

SA among preschool children was the primary outcome variable of this study. It was assessed using an eight‐item dichotomous (yes/no) questionnaire adapted from Leung (2008) [24] and subsequently used in Bangladeshi studies by Mahmud et al. (2020) [25] and Hosen et al. (2021) [26].

Each item was scored as 1 for “yes” and 0 for “no,” resulting in a total score ranging from 0 to 8. Based on established cut‐off criteria, a score of 0–1 was classified as nonaddicted, whereas a score of 2–8 was classified as smartphone addicted.

The independent variables included a range of sociodemographic, familial, child‐related, and behavioral characteristics. These variables are presented in Table 1. Sociodemographic variables included family type, area of residence, maternal age, parental education levels, dual‐income family status, parental occupation, and monthly family income. Child‐related variables included child age, gender, number of siblings, and whether the child owned a personal smartphone.

**Table 1:** Descriptions of study variables and categorizations.

Variable	Categorization
Child age (years)	3–4, 5–6
Child gender	Male, female
Family type	Nuclear, extended
Living place	Rural and semiurban (rurban)
Maternal age (years)	18–25, 26–30, > 30
Mother′s education	Illiterate, primary, secondary, higher secondary or above
Father′s education	Illiterate, primary, secondary, higher secondary or above
Dual‐income family	Yes, no
Mother′s occupation	Housewife, labor/worker, service
Father′s occupation	Business, labor/worker, service
Family monthly income	< 20,000; 20,000–30,000; > 30,000
Child has own smartphone	Yes, no
Number of siblings	Yes, no
PPBTS	More positive, positive, negative, more negative
PDMUS	≤ 3 h, > 3 h
PDCUS	< 1 h, ≥ 1 h

Behavioral and parental variables included parents′ perception and behavior toward smartphone (PPBTS) use, daily smartphone use by the mother (PDMUS), and daily smartphone use by the child (PDCUS). These variables were categorized based on previous literature and conceptual relevance to smartphone use behavior.

Residential status was classified as rural and semiurban (rurban) based on administrative boundaries. Households located within municipal areas were classified as semiurban, whereas those located outside municipal areas were considered rural, consistent with the study′s focus on predominantly rural communities in Noakhali District.

In addition, residential status was classified as semiurban (rurban) and rural. Households located within municipal areas were classified as rurban, whereas those located outside municipal areas were classified as rural, to ensure alignment with the study objectives and the variables used in the analysis.

### 2.5. Questionnaire Development, Validity, and Reliability

The questionnaire was adapted from previously published studies on SA and PSU among young children. To establish content validity, the draft questionnaire was reviewed by experts in public health and statistics, and revisions were made based on their recommendations. A pilot survey involving 30 parents from a neighboring community who were not included in the final study was conducted to assess the clarity, comprehensibility, and feasibility of the questionnaire. Necessary modifications were made before the main survey.

The Smartphone Addiction Scale (SAS) consisted of eight dichotomous (yes/no) items. Internal consistency reliability was evaluated using Cronbach′s alpha. Construct validity was assessed using confirmatory factor analysis (CFA) with the weighted least squares mean and variance adjusted (WLSMV) estimator, which is appropriate for dichotomous variables. Model fit was evaluated using the chi‐square statistic (*χ*2), Comparative Fit Index (CFI), Tucker–Lewis Index (TLI), root mean square error of approximation (RMSEA), and standardized root mean square residual (SRMR). Cronbach′s alpha values of ≥ 0.70 were considered indicative of acceptable internal consistency. Standardized factor loadings were examined to evaluate item performance, with values of approximately ≥ 0.40 generally considered desirable; however, items with lower loadings were retained when supported by their conceptual importance and role in the predefined scoring framework.

### 2.6. Conceptual Framework of the Study

**Figure 1:**
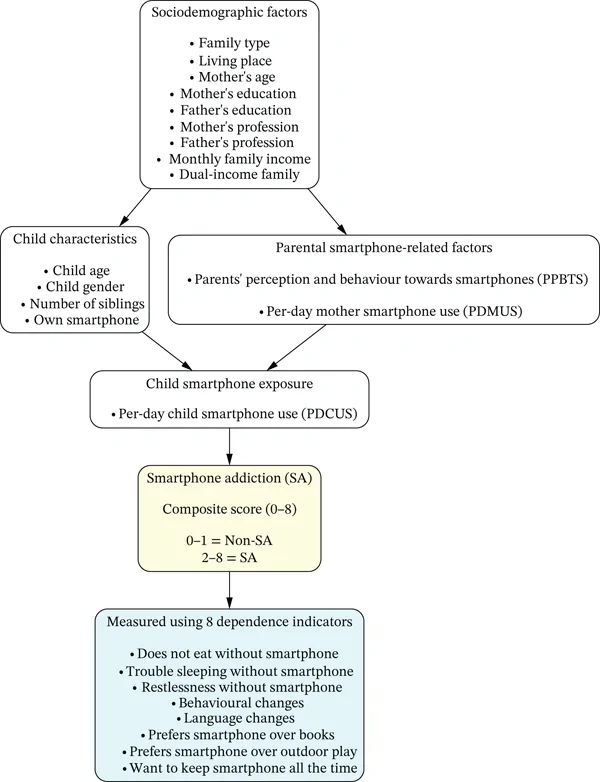
Conceptual framework of the study.

The conceptual framework (Figure 1) was developed based on previous literature and the study objectives to illustrate the hypothesized determinants of SA among preschoolers. It proposes that sociodemographic and familial characteristics, including parental education, occupation, income, smartphone ownership, PPBTS, and parental smartphone use, influence children′s daily smartphone use, which subsequently affects the risk of SA. Guided by this framework, the associations between these factors and SA were examined using chi‐square tests, CFA, correlation analysis, and multivariable binary logistic regression.

### 2.7. Statistical Analysis

Descriptive statistics were used to summarize the characteristics of the study participants and study variables. Frequency distributions and percentages were computed for categorical variables.

In the bivariate analysis, the association between SA and each independent variable was examined using the chi‐square test. Variables with a statistically significant association (*p* < 0.05) were considered for further analysis.

A multivariable binary logistic regression model was then performed to identify factors independently associated with SA while controlling for other covariates. Adjusted odds ratios (AORs) with 95% confidence intervals (CIs) were reported to measure the strength of association between predictors and SA.

Confounding was addressed by including variables with a *p* − value < 0.05 in the bivariate analysis together with variables identified a priori from previous literature as potential confounders. These variables included child age, child sex, parental education, parental occupation, family monthly income, child smartphone ownership, parental daily smartphone use, and PPBTS. A multivariable binary logistic regression model was then fitted to estimate AORs while controlling for these potential confounders. Although several important confounders were adjusted for, residual confounding cannot be completely excluded because of the observational cross‐sectional design of the study.

Multicollinearity among independent variables was assessed using tolerance values and the variance inflation factor (VIF). Tolerance values greater than 0.20 and VIF values less than 5 were considered indicative of no significant multicollinearity.

Interaction effects between independent variables were not formally assessed because of model parsimony considerations and sample size limitations.

All statistical analyses were performed using the Statistical Package for Social Sciences (IBM SPSS, Version 25.0) and R statistical software (version 4.1.2). A two‐sided *p* value of < 0.05 was considered statistically significance.

## 3. Results

### 3.1. Reliability and Construct Validity of the SA Scale

The internal consistency (Table 2) of the eight‐item SAS was assessed using Cronbach′s alpha. The scale demonstrated good internal consistency (Cronbach′s *α* = 0.82).

**Table 2:** Internal consistency reliability of the Smartphone Addiction Scale (SAS).

Scale	Number of items	Cronbach′s *α*	Interpretation
Smartphone Addiction Scale (SAS)	8	0.82	Good

Corrected item‐total correlations ranged from 0.11 to 0.80 (Table 3). Although Item 1 showed a relatively low corrected item‐total correlation (0.11), it was retained to preserve the conceptual coverage and predefined scoring system of the instrument. Deleting Item 1 increased Cronbach′s alpha to 0.85; however, the overall reliability of the complete 8‐item scale remained acceptable.

**Table 3:** Item analysis of the Smartphone Addiction Scale (SAS).

Item	Corrected item‐total correlation	Cronbach′s *α* if item deleted
Item_1_	0.11	0.85
Item_2_	0.41	0.81
Item_3_	0.80	0.76
Item_4_	0.59	0.79
Item_5_	0.55	0.80
Item_6_	0.53	0.80
Item_7_	0.74	0.77
Item_8_	0.62	0.78

Construct validity of the SAS was evaluated using CFA. The hypothesized one‐factor model demonstrated excellent fit to the observed data (*χ* ^2^ = 36.90, df = 20, *χ* ^2^/df = 1.85, CFI = 0.997, TLI = 0.996, RMSEA = 0.046, and SRMR = 0.069). All fit indices satisfied the recommended criteria, indicating that the proposed measurement model adequately represented the underlying construct (Table 4).

**Table 4:** Confirmatory factor analysis model fit.

Fit index	Obtained value	Recommended value	Interpretation
*χ* ^2^	36.90	—	—
Degrees of freedom	20	—	—
*χ* ^2^/df	1.85	< 3.00	Good
CFI	0.997	≥ 0.95	Excellent
TLI	0.996	≥ 0.95	Excellent
RMSEA	0.046	< 0.08	Good
SRMR	0.069	< 0.08	Acceptable

Construct validity was evaluated using CFA with the WLSMV estimator (Table 5). The one‐factor model demonstrated good model fit (*χ* ^2^ = 36.90, df = 20, *χ* ^2^/df = 1.85, CFI = 0.997, TLI = 0.996, RMSEA = 0.046, SRMR = 0.069). Standardized factor loadings ranged from 0.176 to 0.999. Although Item 1 exhibited a relatively low loading (0.176), the remaining items showed moderate to very strong loadings (0.633–0.999), supporting the unidimensional structure of the SAS.

**Table 5:** Standardized factor loadings.

Item	Standardized loading
Item_1_	0.176
Item_2_	0.633
Item_3_	0.999
Item_4_	0.811
Item_5_	0.766
Item_6_	0.741
Item_7_	0.928
Item_8_	0.845

### 3.2. Sociodemographic and Familial Characteristics

Table 6 shows that among the 441 respondents, 50.6% are male preschoolers and 49.4% are female preschoolers. About 73% of Childs′ mothers are housewives, whereas most of the fathers (42%) are in jobs/services. 54.6% of households live in rurban areas, and 45.4% in rural areas.

**Table 6:** Frequency distribution of the sociodemographic and familial characteristics of the respondents.

Variable	Category	Frequency	Percentage (%)
Family type	Nuclear	249	56.5
	Extended	192	43.5

Living place	Rural	200	45.4
	Semiurban (rurban)	241	54.6

Mothers age	18–25	113	25.6
	26–30	214	48.5
	> 30	114	25.9

Mother′s education level	Illiterate	87	19.7
	Primary	114	25.9
	Secondary	104	23.6
	Higher secondary or above	136	30.8

Father′s education level	Illiterate	53	12.0
	Primary	73	16.6
	Secondary	78	17.7
	Higher secondary or above	237	53.7

Dual‐income family	Yes	149	33.8
	No	292	66.2

Mother′s profession	Housewife	322	73
	Labor/worker	44	10.0
	Job/service	75	17.0

Father′s profession	Business	138	31.3
	Labor/worker	120	27.2
	Job/service	183	41.5

Family monthly income	< 20,000	118	26.8
	20,000–30,000	174	39.5
	≥ 30,000	149	33.8

Child have own smartphone	Yes	149	33.8
	No	292	66.2

Child age	1‐less than 3 years	218	49.4
	3–5 years	223	50.6

Child gender	Male	223	50.6
	Female	218	49.4

Number of siblings	Yes	379	14.1
	No	62	85.9

PPBTS	More positive	46	10.4
	Positive	170	38.5
	Negative	142	32.3
	More negative	83	18.8

PDMUS	≤ 3 h	251	56.9
	> 3 h	190	43.1

PDCUS	< 1 h	168	38.1
	> 1 h	273	61.9

Abbreviations: PPBTS, parents′ perception and behavior toward smartphone; PDCUS, per day child use smartphone (h); and PDMUS, per day mother use the smartphone (h).

Most children (85.9%) had no siblings. More than half of the mothers (56.9%) used smartphones for ≤ 3 h per day, whereas 61.9% of children used smartphones for ≥ 1 h per day. In terms of parental attitudes, the largest proportion of parents (38.5%) reported positive perceptions and behaviors toward smartphone use, whereas 32.3% reported negative perceptions.

**Figure 2:**
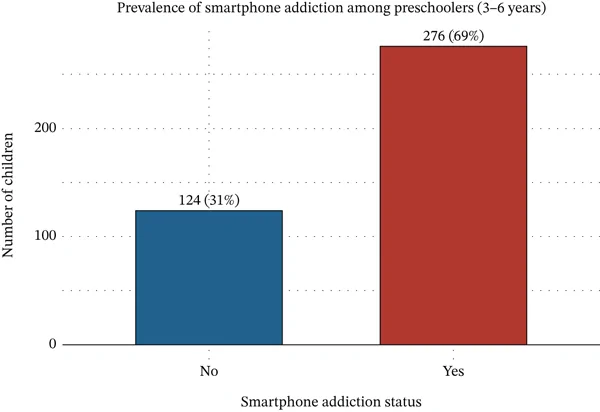
Smartphone addiction among preschoolers (3–6 years).

Overall, 69.0% of preschool children were classified as having SA (Figure 2), with a slightly higher proportion among males (52.4%) compared with females (47.6%).

### 3.3. Association Between SA and Independent Variables

Table 7 presents the results of the chi‐square tests examining the association between SA and selected sociodemographic and familial variables. Significant associations with SA were observed for family type, maternal age, dual‐income family status, father′s occupation, ownership of a personal smartphone, number of siblings, PPBTS, daily maternal smartphone use (PDMUS), and daily child smartphone use (PDCUS) (*p* < 0.05).

**Table 7:** Association of smartphone addiction with selected variables.

Variables		Smartphone addiction		Value of test statistic	*p*
		Yes	No	*χ*2	
Family type	Nuclear	187	62	9.459	0.002
	Extended	118	74		

Age of mother	18–25	95	18	15.835	0.000
	26–30	137	77		
	> 30	73	41		

Dual‐income family	Yes	114	35	5.689	0.017
	No	191	101		

Father′s Profession	Business	97	41	8.861	0.012
	Labor/worker	94	26		
	Job/service	114	69		

Child have own smartphone	Yes	144	5	79.693	0.000
	No	161	131		

Number of siblings	Yes	270	109	13.088	0.023
	No	35	27		

Parents perception and behavior toward smartphone	More positive	42	4	140.438	0.000
	Positive	158	12		
	Negative	85	57		
	More negative	20	63		

Per day mother use the smartphone	≤ 3 h	145	106	35.45	0.000
	> 3 h	160	30		

Child use smartphone (h)	< 1 h	64	104	122.80	0.000
	> 1 h	241	32		

Abbreviations: PPBTS, parents′ perception and behavior toward smartphone; PDCUS, per day child use smartphone (h); and PDMUS, per day mother use the smartphone (h).

No statistically significant associations were observed for residential status, child age, child gender, maternal education, father′s education, or family monthly income (*p* > 0.05).

### 3.4. Multicollinearity Diagnostics

Multicollinearity among the independent variables was assessed using tolerance and variance VIF. Tolerance values ranged from 0.384 to 0.865, whereas VIF values ranged from 1.156 to 2.605 (Table 8). All VIF values were below three, indicating no evidence of problematic multicollinearity among the predictor variables.

**Table 8:** Collinearity diagnostics of the independent variables.

Variable	Tolerance	VIF
Family type	0.770	1.299
Living place	0.611	1.636
Age of mother	0.730	1.369
Mother′s education level	0.470	2.127
Father′s education level	0.474	2.108
Dual‐income family	0.401	2.494
Mother′s profession	0.384	2.605
Father′s profession	0.799	1.252
Family monthly income	0.527	1.899
Child has own smartphone	0.682	1.467
Child age	0.865	1.156
Child gender	0.841	1.189
Number of siblings	0.711	1.406
Parents′ perception and behavior toward smartphone	0.542	1.844
Mother′s daily smartphone use	0.604	1.656
Child′s smartphone use (h/day)	0.588	1.700

*Note:* All VIF values were below three, indicating no evidence of multicollinearity among the predictor variables.

Abbreviation: VIF, variance inflation factor.

### 3.5. Multivariable Logistic Regression Analysis

Table 9 shows that, after adjusting for potential confounders, father′s occupation, child ownership of a smartphone, parental perception and behavior toward smartphones, and daily child smartphone use remained statistically significant predictors of SA.

**Table 9:** Binary logistic regression analysis of associations of sociodemographic and economic characteristics and parental attitudes with smartphone addiction, N = 441.

Variable	Category	Coefficient	*p*	AOR	95% C.I for AOR	
					Lower	Upper
Family type	Nuclear	Ref	Ref	Ref	Ref	Ref
	Extended	−0.057	0.867	0.945	0.484	1.844

Age of mother	18‐25	Ref	0.170	Ref	Ref	Ref
			Ref			
	26−30	−0.270	0.539	0.763	0.323	1.806
	> 30	0.417	0.223	1.517	0.775	2.970

Dual‐income family	Yes	Ref	Ref	Ref	Ref	Ref
	No	0.027	0.934	1.028	0.534	1.978

Father′s profession	Business	Ref	0.005	Ref	Ref	Ref
			Ref			
	Labor/worker	−0.527	0.149	0.591	0.289	1.207
	Job/service	−1.310	0.001	0.270	0.123	0.592

Child have own smartphone	Yes	Ref	Ref	Ref	Ref	Ref
	No	−2.539	0	0.079	0.027	0.230

Number of siblings		−0.236	0.149	0.790	0.573	1.088

PPBTS	More positive	Ref	0	Ref	Ref	Ref
			Ref			
	Positive	−1.515	0.039	0.220	0.052	0.925
	Negative	−2.680	0	0.069	0.026	0.181
	More negative	−1.475	0	0.229	0.103	0.510

PDMUS	≤ 3 h	Ref	Ref	Ref	Ref	Ref
	> 3 h	‐0.426	0.231	0.653	0.325	1.311

PDCUS	< 1 h	Ref	Ref	Ref	Ref	Ref
	> 1 h	1.689	0	5.416	2.800	10.476

Constant		1.239	0.041	3.619		

Abbreviations: PPBTS, parents′ perception and behavior toward smartphone; PDCUS, per day child use smartphone (h); and PDMUS, per day mother use the smartphone (h).

Children whose fathers were engaged in service or job‐related occupations had significantly lower odds of SA compared with those whose fathers were involved in business (AOR = 0.27, 95% CI: 0.123–0.592). Children who did not own a smartphone had significantly lower odds of SA compared with those who owned a personal device (AOR = 0.079, 95% CI: 0.027–0.230).

Parental perceptions and behaviors regarding smartphone use were strongly associated with SA. Compared with children whose parents reported more positive attitudes, those with negative perceptions had significantly lower odds of SA (AOR = 0.069, 95% CI: 0.026–0.181).

Children who used smartphones for more than 1 h per day were more likely to have SA compared to those using smartphones for less than 1 h per day (AOR = 5.416, 95% CI: 2.800–10.476).

Table 10 presents the model diagnostics for the logistic regression analysis. The Hosmer–Lemeshow goodness‐of‐fit test indicated an acceptable model fit (*χ* ^2^, *d* *f* = 8, *p* = 0.725). The Nagelkerke R‐squared value of 0.587 suggests that approximately 58.6% of the variation in SA was explained by the variables included in the model.

**Table 10:** Model summary and Hosmer–Lemeshow test.

Nagelkerke R square	Hosmer–Lemeshow test		
	*Chi-square*	*Df*	*p*
0.586	5.297	8	0.725

## 4. Discussion

The present study examined the prevalence of SA and its associated factors among preschool children in Noakhali District, a predominantly rural district of Bangladesh. The findings indicate that 69% of preschool children met the criteria for SA, suggesting that PSU is common even among very young children in this setting. Although this prevalence is lower than the 86% reported among preschool children in Dhaka [8], it remains substantially higher than estimates reported in other countries, such as South Korea, where the prevalence of PSU among preschoolers was 17% [10]. Differences in prevalence across studies may reflect variations in study populations, measurement instruments, age groups, parental supervision, and sociocultural contexts. Furthermore, previous studies among older populations have reported high levels of PSU, including a prevalence of 87% among students [27], indicating that excessive smartphone use is a growing public health concern across age groups. By focusing on a predominantly rural district, the present study extends the existing evidence beyond metropolitan settings. It suggests that SA is not limited to highly urbanized areas of Bangladesh.

The present study identified father′s occupation as an independent factor associated with preschool children′s SA. Specifically, children whose fathers were employed in service‐related occupations had significantly lower odds of SA than those whose fathers were engaged in business. Although the underlying mechanisms cannot be established because of the cross‐sectional design, this finding may reflect differences in work schedules, parental supervision, and family routines that influence children′s access to smartphones. In some households, smartphones may also be used to occupy children while parents are working or handling household responsibilities, thereby increasing children′s exposure to digital devices. Similar associations have been reported in previous studies, which identified parental occupation, parental smartphone use, educational attainment, maternal age, and family income as important correlates of PSU among preschool children [8, 27]. Other studies have likewise suggested that parental occupational characteristics and family environments may influence young children′s digital media use and problematic smartphone behaviors [8, 28].

Personal smartphone ownership was also strongly associated with SA. Preschool children who did not own a smartphone had substantially lower odds of SA than those who owned one. This finding is consistent with previous studies demonstrating that personal ownership of smartphones increases opportunities for frequent and unsupervised use, thereby increasing the likelihood of problematic smartphone behaviors [29, 30]. Although ownership itself may not directly cause addiction, it likely facilitates greater accessibility and prolonged exposure, particularly when parental monitoring is limited.

Parents′ perceptions and behaviors toward smartphone use were another important factor associated with SA. Children whose parents reported more negative perceptions of smartphone use had significantly lower odds of SA than those whose parents reported more positive perceptions. This finding supports previous research indicating that parenting practices, parental attitudes, and parental smartphone behaviors play important roles in shaping children′s digital media habits [3, 31]. Rather than suggesting that negative attitudes alone prevent SA, these findings may indicate that parents who are more aware of the potential adverse effects of excessive smartphone use are more likely to establish rules, supervise screen time, and encourage alternative activities. Consequently, parental awareness and consistent monitoring may be important components of strategies to reduce PSU among preschool children.

Daily smartphone use was the strongest behavioral factor associated with SA. Children who used smartphones for more than 1 h per day had more than five times the odds of SA than those who used them for less than 1 h per day. This finding is consistent with previous studies reporting that the risk of PSU increases with longer daily screen time [10]. Similar studies have shown that children with PSU spend considerably more time using smartphones and are at substantially greater risk of addiction than those with lower usage durations [32]. Moreover, studies conducted in different populations have consistently reported positive associations between increased daily smartphone use and problematic smartphone behaviors, despite differences in age groups and screen‐time thresholds [8, 10, 12, 33]. Collectively, these findings suggest that prolonged daily smartphone exposure is a key behavioral correlate of SA among preschool children. From a public health perspective, interventions that promote age‐appropriate screen‐time limits, encourage parental supervision, and provide alternative recreational activities may help reduce excessive smartphone use during early childhood.

## 5. Limitations of the Study

This study has several limitations that should be considered when interpreting the findings. First, the cross‐sectional design precludes any inference of causal relationships between SA and the associated factors. Second, although a multistage sampling approach was initiated, convenience sampling may have introduced selection bias and affected the sample′s representativeness, so our findings should be interpreted with caution and not extended to different populations. Third, the study was conducted in selected areas of the Noakhali district; therefore, the results may not be generalizable to all preschool children in Bangladesh or to other settings with different socio‐demographic characteristics. Fourth, data were collected through parent‐reported responses, which may be subject to recall bias and social desirability bias.

Fifth, although multivariable logistic regression was used to adjust for several factors, residual confounding cannot be ruled out given the observational nature of the study and the variable selection approach. Additionally, interaction effects between variables were not formally assessed, which may limit a more comprehensive understanding of the relationships among predictors.

Despite these limitations, this study provides important preliminary evidence on the prevalence of SA among preschool children in a rural context and highlights key demographic and family‐related factors. These findings can inform future research using more rigorous sampling strategies and longitudinal designs to understand causal pathways and context‐specific influences better.

## 6. Conclusion

This study found a high prevalence of SA among preschool children in Noakhali district, Bangladesh, with approximately seven in 10 children meeting the study criteria for SA. Several family and child‐related factors, including father′s occupation, child ownership of a smartphone, parents′ perceptions and behaviors toward smartphone use, and daily smartphone use, were independently associated with SA. These findings suggest that the family environment and children′s smartphone use patterns play important roles in SA during early childhood. Promoting age‐appropriate screen‐time limits, delaying personal smartphone ownership, and increasing parental awareness and supervision may help reduce excessive smartphone use among preschool children.

As this study was conducted in a predominantly rural district of Bangladesh, the findings contribute context‐specific evidence from an underrepresented population. However, they should be interpreted in light of the study′s methodological limitations and should not be generalized beyond similar settings. Future studies using longitudinal designs, probability‐based sampling, and nationally representative populations are needed to confirm these findings, examine causal pathways, and evaluate interventions to prevent SA among young children.

## Author Contributions

Kabir Hossain: Conceptualization, data curation, formal analysis, software, writing—original draft, writing—review and editing. Md. Mamun Miah: Conceptualization, supervision, methodology, resources, project administration, software, validation, writing—original draft, writing—review and editing. Najma Begum: Visualization, writing—review and editing.

## Funding

No funding was received for this manuscript.

## Ethics Statement

The study was conducted in accordance with the ethical principles of research. This study obtained ethical approval from the Noakhali Science and Technology University Ethical Committee (NSTUEC) with reference number NSTU/SCI/EC/2023/145(A).

## Conflicts of Interest

The authors declare no conflicts of interest.

## Data Availability

The data that support the findings of this study are available from the corresponding author upon reasonable request.
